# A Deep-Learning-Computed Cancer Score for the Identification of Human Hepatocellular Carcinoma Area Based on a Six-Colour Multiplex Immunofluorescence Panel

**DOI:** 10.3390/cells12071074

**Published:** 2023-04-02

**Authors:** Axel Dievernich, Johannes Stegmaier, Pascal Achenbach, Svetlana Warkentin, Till Braunschweig, Ulf Peter Neumann, Uwe Klinge

**Affiliations:** 1 Department of General, Visceral and Transplant Surgery, University Hospital RWTH Aachen, 52074 Aachen, Germany; 2 Forschungs-und Entwicklungsgesellschaft FEG Textiltechnik, 52070 Aachen, Germany; 3 Institute of Imaging and Computer Vision, RWTH Aachen University, 52074 Aachen, Germany; 4 Department of Neurology, University Hospital RWTH Aachen, 52074 Aachen, Germany; 5 Institute of Neuropathology, University Hospital RWTH Aachen, 52074 Aachen, Germany; 6 Institute of Pathology, University Hospital RWTH Aachen, 52074 Aachen, Germany; 7 Department of Surgery, Maastricht University Medical Center+, 6229 HX Maastricht, The Netherlands

**Keywords:** hepatocellular carcinoma, HCC, convolutional neural network, CNN, U-net, multiplex, fluorescence, immunofluorescence, microscopy, cancer score

## Abstract

Liver cancer is one of the most frequently diagnosed and fatal cancers worldwide, with hepatocellular carcinoma (HCC) being the most common primary liver cancer. Hundreds of studies involving thousands of patients have now been analysed across different cancer types, including HCC, regarding the effects of immune infiltrates on the prognosis of cancer patients. However, for these analyses, an unambiguous delineation of the cancer area is paramount, which is difficult due to the strong heterogeneity and considerable inter-operator variability induced by qualitative visual assessment and manual assignment. Nowadays, however, multiplex analyses allow the simultaneous evaluation of multiple protein markers, which, in conjunction with recent machine learning approaches, may offer great potential for the objective, enhanced identification of cancer areas with further in situ analysis of prognostic immune parameters. In this study, we, therefore, used an exemplary five-marker multiplex immunofluorescence panel of commonly studied markers for prognosis (CD3 T, CD4 T helper, CD8 cytotoxic T, FoxP3 regulatory T, and PD-L1) and DAPI to assess which analytical approach is best suited to combine morphological and immunohistochemical data into a cancer score to identify the cancer area that best matches an independent pathologist’s assignment. For each cell, a total of 68 individual cell features were determined, which were used as input for 4 different approaches for computing a cancer score: a correlation-based selection of individual cell features, a MANOVA-based selection of features, a multilayer perceptron, and a convolutional neural network (a U-net). Accuracy was used to evaluate performance. With a mean accuracy of 75%, the U-net was best capable of identifying the cancer area. Although individual cell features showed a strong heterogeneity between patients, the spatial representations obtained with the computed cancer scores delineate HCC well from non-cancer liver tissues. Future analyses with larger sample sizes will help to improve the model and enable direct, in-depth investigations of prognostic parameters, ultimately enabling precision medicine.

## 1. Introduction

In 2018, liver cancer was the 7th most commonly diagnosed cancer (4.7%) and the 3rd most common cancer leading to death (8.2%) worldwide [1]. Hepatocellular carcinoma (HCC) is the most frequent primary liver cancer and usually occurs in patients secondary to chronic hepatitis or cirrhosis resulting from hepatitis virus infection, or from non-virus related causes such as high alcohol consumption and non-alcoholic fatty liver disease [2,3,4].

Various immunohistochemical markers, representing distinct immune cell types or functional properties/characteristics, are currently used to characterise cancer landscapes to predict clinical outcomes or responses to therapy [5]. The prerequisite for any analysis is a clear determination of the cancer area to be delineated from adjacent non-cancer tissues. An unambiguous assignment is difficult, as cancer landscapes usually exhibit marked heterogeneity, but it should be possible with the help of high-content imaging, recent machine learning approaches such as multilayer perceptrons (MLPs), or convolutional neural networks (CNNs), and corresponding expert annotations. However, a strict dichotomous separation between cancer and non-cancer tissues may disregard crucial intermediate forms with incomplete manifestations of cancer features expressed in variable marker intensities throughout the cancer area and/or the parallel analysis of multiple markers. Multiplex immunofluorescence staining is particularly well-suited for the latter and offers the additional advantage that the same tissue sections and markers can be used beyond cancer classification for further in situ analyses of immune parameters related to, for example, patient survival. The question, therefore, arises as to which analytical approach/model is best suited to combine morphological and immunohistochemical data from a five-marker multiplex immunofluorescence (mIF) panel and nuclear counterstain DAPI into a cancer score to automatically identify the cancer area that best matches an independent pathologist’s judgement/assignment.

An exemplary mIF panel of commonly studied markers for the prognosis of patients with cancers, consisting of four lymphocyte markers, CD3 for T cells, CD4 for T helper cells, CD8 for cytotoxic T cells, and FoxP3 for regulatory T cells, and the checkpoint protein programmed cell death ligand 1 (PD-L1), which is mainly found on immune cells and inhibits T cell responses, was used [5]. DAPI was used to reliably detect nuclei and determine their intensity as an indicator of abnormal DNA content (aneuploidy); the standard deviation of the intensity of all pixels contained in DAPI-based segmentation masks was used to determine heterogeneity as an indicator of genetic instability; and various morphological features, such as nuclear sizes (i.e., nuclear areas), centroids, eccentricities, minor and major axis lengths, among others, were used [6,7,8,9]. In addition, nuclear areas were used as a basis for measuring marker intensities at the cellular level and for determining marker heterogeneity in the vicinity of individual cells. In this way, a total of 68 cell-specific features were determined for each cell.

For consistency with the independent pathologists’ judgements/assignments, we tested four different approaches/models:

(1) First, we tested a correlation-based selection of individual cell features (ICFs) by calculating the absolute correlation value of each feature with the pathologists’ assignment and then combining the five features with the highest correlations into a cancer score.
(2) We then tested a multivariate analysis of variance (MANOVA)-based selection of features, combining only the features with the highest correlations with the pathologist’s assignment and those with the lowest correlations with the previously selected features to calculate the cancer score.
(3) Next, we tested an MLP, a feedforward artificial neural network trained with all the features as input and formulated the computation of the cancer score as a regression task. As the MLP is trained on individual cells, the context is only taken into account indirectly.
(4) Finally, we trained a U-net, a CNN architecture specifically designed for semantic biomedical image segmentation, which also takes into account the spatial context [10].

## 2. Materials and Methods

We analysed sections of archived formalin-fixated, paraffin-embedded (FFPE) tissues from 12 patients surgically treated for hepatocellular carcinoma (HCC) from our tissue bank (approval by the Ethics Committee EK 106/18). Electronic medical records were used for the characteristics of the cohort (Table 1).

**Figure 1:**
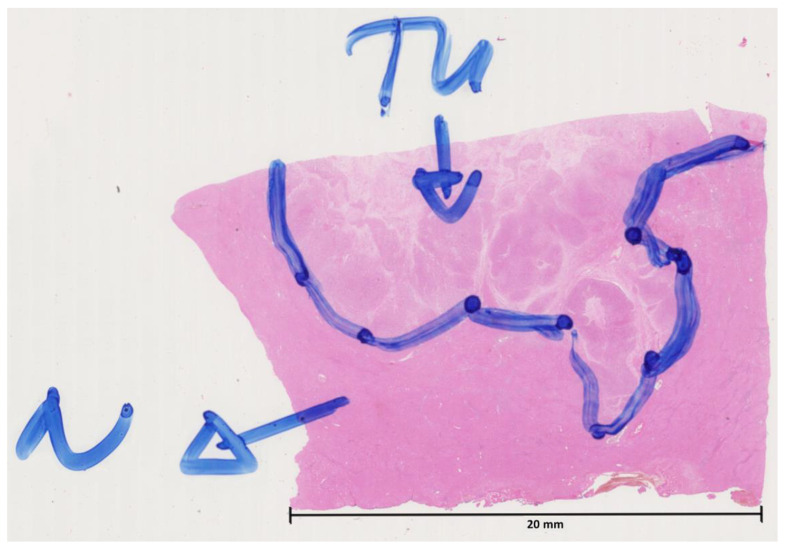
Digitised haematoxylin-and-eosin-stained tissue section with manually assigned cancer area (TU) and non-tumour (N) liver tissue by an independent pathologist.

Prior to immunofluorescence staining, at least one section of each HCC sample was stained with haematoxylin and eosin (H&E). The H&E sections were digitised and submitted to two independent pathologists for marking the cancer area (Figure 1). The respective annotated regions were used to create masks for the cancer area of the primary tumour and the adjacent/surrounding non-tumour liver tissues in the subsequently multiplex-stained tissue sections.

### 2.1. Immunofluorescence Staining

#### 2.1.1. General

All steps were performed at room temperature. Serial 2 µm sections of each HCC specimen were cut using a manually operated rotary microtome with blades for harder tissues. The sections were first labelled with the pan T lymphocyte (T cell) marker CD3 followed by CD4 for T helper cells, PD-L1 for programmed cell death ligand 1, CD8 for cytotoxic T cells, and finally FoxP3 for regulatory T cells (Table 2). Opal^TM^ 480 was used as the fluorophore for CD3, Opal^TM^ 520 for CD4, Opal^TM^ 620 for CD8, Opal^TM^ 690 for PD-L1, and Opal^TM^ 780 for FoxP3. Opal^TM^ reagents were acquired from Akoya Biosciences, Marlborough, MA, USA. All antibodies were diluted with Antibody Diluent (with background reducing components; Dako, Düsseldorf, Germany). Secondary antibodies were applied with the ImmPRESS^®^ HRP Polymer Detection Kit (Vector Laboratories, Burlingame, CA, USA). Fluorophores were diluted with 1× Plus Manual Amplification Diluent (Akoya Biosciences, Marlborough, MA, USA). DAPI was used as a nuclear counterstain.

#### 2.1.2. Protocol

A similar protocol to that used for the other 5-marker multiplex immunofluorescence staining panels and the nuclear counter stain DAPI has already been described in detail [10]. Briefly, tissue sections were deparaffinised and rehydrated before incubation in formalin. Then, antigens were retrieved. Sections were washed and cooled before non-specific binding was blocked. These steps were followed by incubation with the primary antibody of the first marker followed by incubation with the secondary antibody. Then, the first fluorophore was applied. Afterwards, tissue sections were microwave-treated for antibody stripping before being cooled and washed. Non-specific binding was blocked again before the primary antibody of the second marker was applied. Sections were rinsed and incubated with the secondary antibody before applying the second fluorophore. The tissue sections were then microwave-treated again for antibody stripping before being cooled and rinsed.

Subsequent markers were applied in the same way as with the second marker. Finally, after the fifth staining cycle, i.e., after the application of the fifth marker, all tissue sections were counterstained with DAPI and cover-slipped.

### 2.2. Immunofluorescence Staining

**Figure 2:**
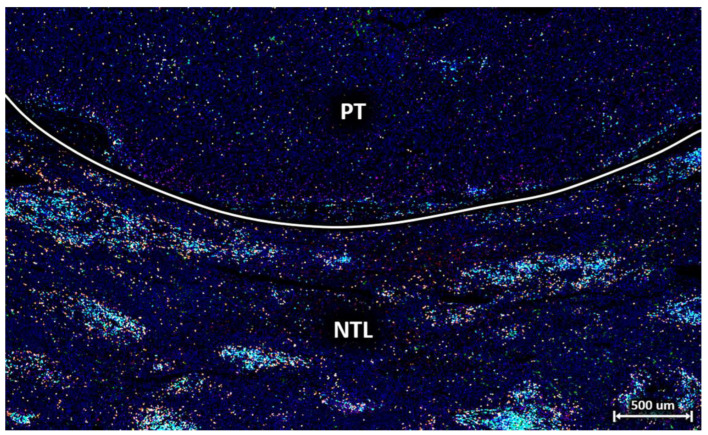
Exemplary section of a multiplex-stained tissue sample with HCC primary tumour (PT) tissue on top and non-tumour liver (NTL) tissue on the bottom. Labelling for nuclei with DAPI (blue), T cells with CD3 (turquoise), T helper cells with CD4 (green), cytotoxic T cells with CD8 (orange), PD-L1 (red), and regulatory T cells with FoxP3 (magenta). The liver parenchyma in the vicinity of the HCC contains several clusters of T cells consisting mainly of T helper cells, cytotoxic T cells, and regulatory T cells.

Fluorescence imaging was performed using the TissueFAXS PLUS Scanning System (TissueGnostics, Vienna, Austria), which consists of an Axio Imager 2 epifluorescence microscope (20×, ZEISS, Oberkochen, Germany) with a motorised reflector turret and high-precision stage for scanning whole slides. The light source used was the Colibri 7 (ZEISS, Oberkochen, Germany), which contains six LED modules and seven fluorescence channels, each of which generates monochromatic light with different wavelengths. LED-optimised filters combined with direct coupling increase sensitivity and ensure optimal excitation and emission spectra. After acquisition, all images were visually checked for staining pattern plausibility using TissueFAXS (Figure 2). The individual tiles of the raw images were then exported for further processing and quantitative analysis using a newly developed software tool we call HCCMiner. HCCMiner is an extension to the open-source MATLAB toolbox SciXMiner [11] and can be downloaded free of charge via the following link: https://github.com/stegmaierj/HCCMiner (accessed on 9 July 2021).

Using HCCMiner, we first used the DAPI images to detect and segment the cell nuclei, the areas of which were recorded and used to measure the mean staining intensities of the fluorophore of each biomarker. Processing was performed for each image tile individually, and we used the publicly available Cellpose algorithm for the nucleus segmentation [12]. As the processing of all tiles was performed in parallel, we subsequently needed to fuse the tile-based results to a consistent representation. The rough alignment of the tiles was already known from the grid layout and acquisition scheme used in TissueFAXS. However, there were small intentional overlaps between adjacent tiles used for the refined alignment. A multi-resolution grid search strategy was used to determine the optimal alignment that minimised the nearest-neighbour distances. Redundant detections in regions of overlapping tiles were removed, i.e., only one detection per segmented nucleus was preserved. The segmented nucleus areas were then used to extract intensity and morphological features (Table S1). For each of the immunohistochemical markers, we considered a cell to be “positive” if the mean intensity for that particular marker was two standard deviations above the mean expression of all cells for that marker. In addition, the centroids of the detected cells, the lengths of the minor and major axes of the nuclei, and the number of “positive” cells within a radius of 300 pixels (~100 µm) around the nucleus of each cell were determined, among other features (Table S1). For each individual cell, all determined data were stored in a unique feature vector together with the independent pathologists’ pre-determined binary assignments based on H&E staining. The feature vectors of the cells were used as input for the four different models to identify the cancer area based on the cancer score.

### 2.3. Creation of Models for the Identification of the Cancer Area

To determine the best method for identifying the cancer area in terms of consistency with the assignments made by the independent pathologists, we developed and compared the following four models.

#### 2.3.1. Model Using Correlation-Based Selection of Individual Cell Features

For this model, the correlations of individual cell features (ICFs) with the pathologists’ cancer masks were calculated to select the five features with the highest absolute correlation coefficients. The method was tested with different input data normalisation methods to compensate for potential value range differences in the extracted features between the data sets. In addition to using the plain feature values (no normalisation), we tested a z-score normalisation (z-score) that scales each feature separately to zero mean and unit variance and a feature-specific normalisation. The feature-specific normalisation strategy left already normalised features such as circularity and eccentricity unchanged and used a percentile normalisation for the remaining features, mapping the 0.01th percentile to 0 and the 99.9th percentile to 1, clipping lower and higher values, respectively. We then computed a normalised cancer score (range: 0–1) as a correlation-weighted sum of the selected features. The weights were based on the absolute value of the correlation of each feature divided by the sum of all absolute correlation coefficients of the selected features such that the weights sum up to one. Moreover, each of the selected features was normalised to obtain a normalised cancer score using a percentile-based normalisation and clipping as described above. Negatively correlated features were inverted before being added to the cancer score.

#### 2.3.2. Model Using MANOVA-Based Selection of Individual Cell Features

The multivariate analysis of variance (MANOVA)-based approach uses the same normalisation strategies as described for the ICF model and combines the selected features in the same fashion to a normalised cancer score. However, the approach selects the five features whose combination best separates the cancer area from the non-cancer area. Therefore, this approach does not include redundant features, but instead, cell features that add the greatest added value to the previous combination of selected cell features are selected step by step.

#### 2.3.3. Model Using a Multilayer Perceptron

Next, we developed a multilayer perceptron (MLP) and formulated the computation of the cancer score as a regression task. An artificial neural network was trained with all cell features as input to predict the pathologist assignment. As the network is trained on individual cells, the spatial context is only indirectly considered via precomputed neighbourhood-related features. The network was made up of an input layer with 68 neurons (1 for each of the features), 3 hidden layers with 340 neurons each (5 × 68), and an output layer with a single neuron that was trained with a mean-squared error (MSE) loss. All fully connected layers were followed by a batch normalisation layer and rectified linear unit (ReLU) activation layers. Moreover, we used a mini-batch size of 128, a learning rate of 1 × 10^−5^, and the Adam optimizer. To convert the raw predictions of the MLP to the respective classes, a fixed threshold in the middle of the non-tumour/tumour labels was used (i.e., smaller values were classified as non-tumour liver tissues, whereas larger values were classified as tumours). The training was performed using the raw features and normalised versions of the features as with the ICF method. To accelerate the training, we only used the feature vectors of every 10th cell and trained for only 1 epoch, which due to a large number of data points (~0.5–2.0 million cells/data points per slide) led to identical results to the training with all data points.

#### 2.3.4. Model Using a U-Net

As the final model, we trained a U-net [13], a convolutional neural network architecture specifically designed for biomedical image segmentation, which also considers the spatial context. As the U-net is an image-based approach, we first converted individual cell features into image data and phrased the computation of the cancer score as a semantic segmentation problem, i.e., the network was trained to perform a pixel-wise classification into the classes non-tumour/tumour. The entire marker expression patterns/intensities of the cells (*n* = 6) as well as the cell densities, areas of the cell nuclei, and the lengths of the minor and major axes of the nuclei were used as input for this model, resulting in a 10-channel input image for the U-net. We used convolutional layers with a receptive field of 3 × 3, and stride 1 and 16 feature maps at the highest resolution level. The number of feature maps is doubled after each of the 3 down-sampling steps, which in turn are performed using strided convolutions with a receptive field of 4 and a stride of 2. Each convolutional layer is followed by LeakyReLU activation layer with a slope of 0.2. The network was trained on 256 × 256 pixel (82 × 82 µm) sized patches that were cropped randomly from the full-size images. Thus, in each iteration, a potentially different patch of each slide is used. We used an MSE loss to learn the class labels and performed the training with the Adam optimizer for 5000 epochs with a batch size of 2, selecting the model that obtained the minimum loss from the validation data set.

#### 2.3.5. Training Details

For the ICF, MANOVA, and MLP methods, we used a leave-one-out cross-validation strategy, i.e., for our data set extracted from *n* = 12 tissue slides, we used N-1 different sets for the correlation-based feature selection and to train the MLP, respectively, and computed the reported scores from the held-out test set. For the U-net, an additional independent validation data set was required, and we thus used N-2 data sets for training, one data set for validation, and one for testing. Considering all the possible permutations for *n* = 12 requires the training of 132 (N × (N − 1)) different models. The presented numbers in the remainder of this work are the averages obtained from the respective test data sets.

### 2.4. Creation of Models for the Identification of the Cancer Area

Statistical analyses were performed with MATLAB^®^ (MathWorks, Natick, MA, USA). Statistical significance values between HCC primary tumour tissues and non-tumour liver tissues were determined with the non-parametric Mann–Whitney U test, and *p*-values of < 0.05 were considered statistically significant.

To assess the performance of the respective models and cancer score classifiers, the accuracy (the ratio of correct predictions divided by all predictions), sensitivity, specificity, and precision (positive predictive value) were determined.

## 3. Results

### 3.1. T Cell Characterisation in Primary Tumour and Non-Tumour Tissues of HCC Patients

**Figure 3:**
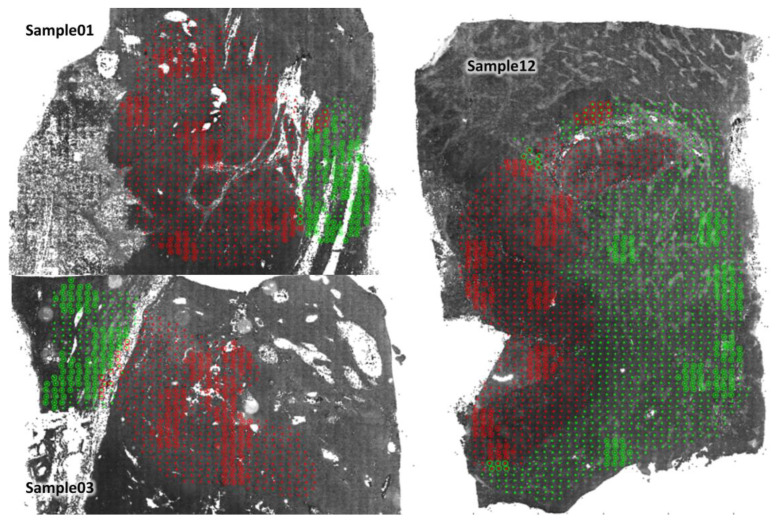
A total of 10 randomly selected virtual biopsies each from the primary tumour (PT) and the non-tumour liver (NTL) tissues with a size of 16 fields of view (16 × 0.15 mm^2^) of 3 HCC patients. Green dots indicate NTL and red dots PT according to the pathologists’ assignment. The field of view classification for the virtual biopsies based on the cancer score of the U-net corresponds to the pathologists’ assignment if the circle has the same colour as the dot in its centre.

To study the T cells in HCC patients, we selected 12 archived tissue sections from patients treated with curative resection who are representative of different tumour stages (Table 1). T cells were quantitatively measured in the primary tumour (PT) and non-tumour liver (NTL) tissues of these patients using HCCMiner. Overall, we obtained data from 15,017,229 cells. A total of 10 randomly selected regions of interest, i.e., virtual biopsies, with a size of 16 fields of view (16 × 0.15 mm^2^) from both PT (~8200 cells) and NTL (~6400 cells) tissues were analysed per sample to determine the mean percentages of “positive” cells for each marker and marker combination (Figure 3). It should be noted that the number of cells in PT tissue is not necessarily higher than that in NTL tissue. The number of cells depends strongly on the selected fields of view and the nature of the contained tissue. For example, there are far fewer cells in desmoplastic or necrotic tumour tissue.

There were significantly fewer CD3^+^ T cells in PT than in NTL tissues (Table 3; 3.9% vs. 8.1%, *p* < 0.001). The same was true for several T cell subsets such as T helper cells (CD3^+^CD4^+^; 0.8% vs. 2.6%, *p* = 0.040) and cytotoxic T cells (CD3^+^CD8^+^; 0.3% vs. 1.2%, *p* = 0.010), but not for CD4^+^ regulatory T cells (FoxP3^+^CD4^+^; 0.3% vs. 0.5%, *p* = 0.750). Interestingly, however, when analysing FoxP3^+^ cells, regardless of the expression of other markers, we found that FoxP3^+^ cells were enriched in the PT tissues compared with the NTL tissues (Table 3; 5.2% vs. 3.1%, *p* = 0.023). Most FoxP3^+^ cells did not express any of the other markers (PT: ~80%, NTL: ~60%). Collectively, these findings highlight the large-scale depletion of T cells and most of their subsets in the PT tissues of HCC patients, with the exception of regulatory T cells.

### 3.2. Individual Cell Features Show Strong Heterogeneity between Patients

**Figure 4:**
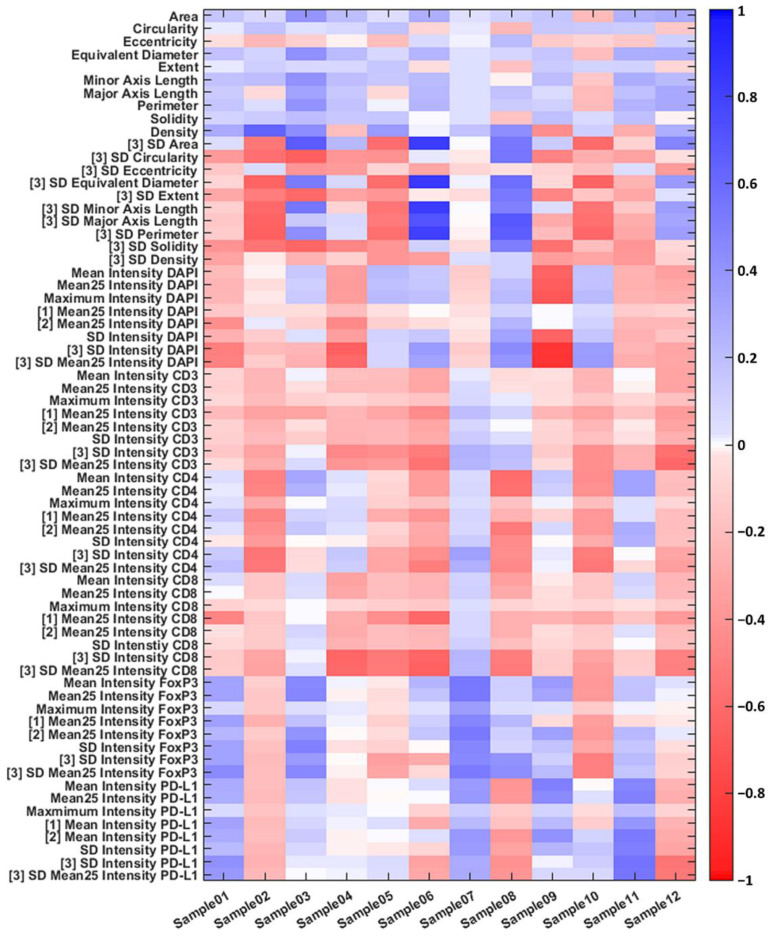
Pearson correlation coefficients of individual cell features with the tumour mask assigned by the pathologists for each HCC sample. Each sample is from a different patient. Dark blue represents a high positive correlation, i.e., the feature for the given HCC sample has a positive correlation with the pathologists’ tumour mask, and dark red represents a negative correlation, i.e., the feature correlates inversely with the pathologists’ assignment. Mean25 = mean of the max. 25% marker intensities, [1] = autofluorescence-corrected marker expression, [2] = autofluorescence-corrected marker expression before counting the number of “positive” cells in a radius of 300 pixels, and [3] = standard deviation (SD) of a feature in the cell’s environment (radius = 300 pixels) as a measure of the amount of variation/heterogeneity.

For each cell, a total of 68 individual cell features (ICFs) were determined, for which the correlation coefficients with the tumour mask were calculated. The correlation coefficients show strong heterogeneity between patients, i.e., there were strong differences up to inverse correlations for a single feature in different patients (Figure 4). Furthermore, in samples 2 and 12, almost all intensity features are negatively correlated with the tumour mask, while in sample 7, most features are positively correlated. Taken together, these findings highlight the inter-patient and intra-tumour heterogeneity as well as the plasticity of HCC cells.

### 3.3. Comparison and Evaluation of the Average Accuracy of the ICF, MANOVA, MLP, and U-Net Models

**Figure 5:**
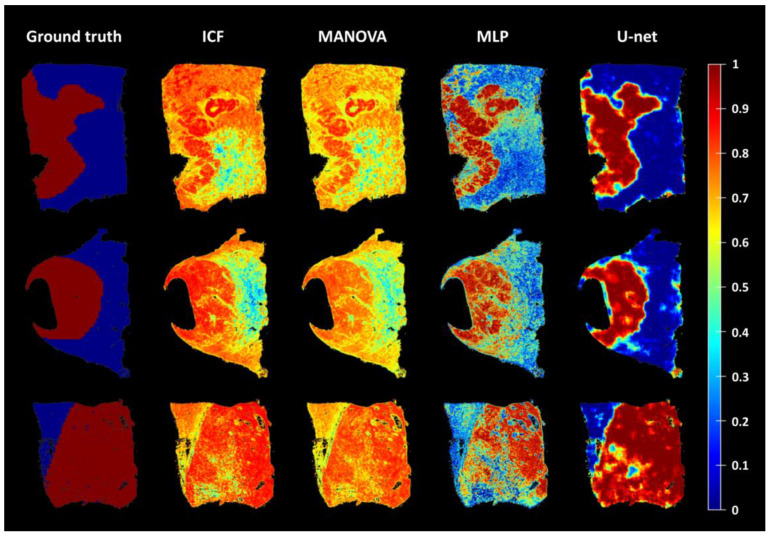
Three examples of tissue sections superimposed with the respective cancer score maps of the different models compared with the assignments (ground truth; red = tumour; blue = non-tumour) by independent pathologists. The cancer score indicates the probability that the cells belong to the tumour tissue, with red representing the highest probability and blue the lowest. ICF = individual cell features, MANOVA = multivariate analysis of variance, and MLP = multilayer perceptron.

To qualitatively compare the four different models for identifying the cancer area, we developed a cancer score (Figure 5). While the ICF and MANOVA models had problems identifying the cancer area, as illustrated by the low contrast of the cancer score maps and no clear delineation of the cancer area, identification was more successful with the MLP and U-net models. Comparing the MLP and the U-net models, the cancer score maps were less well-defined (coarser) for the former. Many areas classified as cancer according to the pathologists’ assignment (ground truth) had a low cancer score indicated by the green or blue colour, and conversely, there were many areas with a high cancer score indicated by the orange or red colour in the assigned non-cancer areas by the pathologists. In addition, many areas in the MLP maps were classified as intermediate between cancer and non-cancer (yellow colour). Consequently, the distinction between cancer and non-cancer in the MLP model was often not as clear/sharp as in the U-net model.

**Figure 6:**
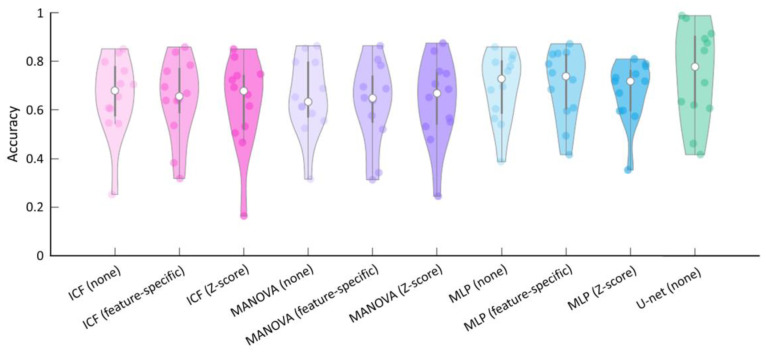
Violin plots showing the accuracies of the individual cell features (ICFs), the multivariate analysis of variance (MANOVA), and the multilayer perceptron (MLP) models with different normalisation methods and the U-net in terms of agreement with the independent pathologists’ assignments. Coloured dots in the violin plots correspond to the individual accuracies of HCC samples. Median accuracies for all samples are indicated by the white dots. Thick grey bars in the centre represent the interquartile ranges.

To quantify the performance of the models and to select the most appropriate model, the accuracies were determined (Table 4, Figure 6). In addition to using the plain feature values (no normalisation), a z-score and feature-specific normalisation were also tested for the ICF, the MANOVA, and the MLP models, while two versions for the U-net were tested: one with the mean marker intensities of the cells (not shown) and one considering the entire marker expression patterns of the cells (raw features). The results show that the performance of the U-net achieved the highest average accuracy of 75% when provided with the raw features. For the ICF, the MANOVA and the MLP models, feature-specific normalisation had little impact on the accuracies, while z-score-based normalisation resulted in lower accuracies in most cases. Based on these results and the spatial representations of the cancer scores (Figure 5), the U-net was selected for further analysis.

### 3.4. Influence of Individual Cell Features on the Accuracy of the U-Net

In order to investigate the influence of individual features on the accuracy in identifying the cancer area, the U-net was trained and validated ten times using eleven samples, omitting a different feature each time (Figure S1). Due to its previously determined high accuracy, the twelfth sample was used for testing. Omitting the nucleus areas yielded the lowest accuracy, followed by the cell densities, and the PD-L1 intensities of the cells. Therefore, surprisingly, the nucleus area feature was most important for identifying the cancer area.

### 3.5. Assessment of the Robustness of the U-Net

The robustness of the U-net in terms of accuracy was assessed by varying the size of the region of interest (ROI) and applying different thresholds for the cancer score to stratify the tissue sections into the primary tumour and non-tumour liver tissues (Figures S2 and S3).

When examining the influence of the size of the ROI, no significant influence was found for sizes up to 25 fields of view (25 × 0.15 mm^2^) (Figure S2, Table S2). However, analyses of the entire tissue sections yielded the highest average accuracy in identifying the cancer area, which underlines the importance of considering as much context as possible. Furthermore, the variance also seemed to increase with the size of the ROIs, which can be explained by a random favourable selection. For example, the ten random ROIs with a size of one field of view were obviously selected more homogeneously compared with the analysis of the whole slide, represented by smaller interquartile ranges and shorter whiskers (Figure S2).

Applying a cancer score threshold to stratify the primary tumour from the non-tumour liver tissues had minimal/no effect on the accuracy (Figure S3). However, the sensitivity, i.e., how many cells within the cancer area are classified as cancer cells, continuously decreased with higher cancer score thresholds, whereas the specificity, i.e., how many cells within the non-tumorous liver tissues are classified as non-cancer cells, continuously increased with higher cancer score thresholds. Consequently, there is a trade-off between sensitivity and specificity, but it must be kept in mind that not all cells within the cancer area are cancer cells and that the cancer areas assigned by pathologists refer to regions, not individual cells.

## 4. Discussion

In this study, a five-marker (four lymphocyte markers and PD-L1) multiplex immunofluorescence panel and nuclear counterstain DAPI were analysed and used to extract cell features to compare four different models for identifying cancer areas in 12 HCC patients and to develop a cancer score. A comparison of the percentage of T cells and their subsets in the primary tumour (PT) tissues with the adjacent non-tumour liver (NTL) tissues showed that in the PT tissues, T cells and most of their subsets, with the exception of regulatory T cells, were depleted to a large extent. Based on the comparison of the four models, the U-net provided the best performance, i.e., the highest accuracy, in terms of consistency with the assigned cancer areas by independent pathologists (75%). For the U-net, basic features such as the areas of the cell nuclei and the cell density within a radius of 300 pixels (~100 µm) around each individual cell had the greatest influence on the accuracy of identifying the cancer area. Depending on the size of the regions of interest examined, the accuracy of the U-net ranged from 66% to 75%, being highest when the entire tissue sections were analysed, which underlines the importance of considering as much context as possible. In addition, the U-net was used to develop a cancer score, which could have great potential in supporting clinical decision making and answering the question of to what extent the expression of specific markers within a tumour has prognostic significance. For the latter issue, the exact cancer area needs to be determined; however, cancer areas are usually marked manually and are thus subject to inter-operator variability induced by qualitative assessment. The application of different thresholds for the cancer score had only a minor impact on the overall accuracy of the U-net, but the sensitivity decreased continuously, and the specificity increased continuously with higher thresholds.

There are only a few other studies which have investigated the identification of cancer cells [8,9] or cancer areas in HCC [14]. Most studies used hyperspectral imaging with or without DAPI, which does not allow for subsequent analyses of immune markers [8,9,14,15]. In addition, some studies have determined the accuracy of HCC differentiation according to the WHO tumour grade based on label-free approaches, but these studies did not identify the cancer areas [14,16]. In a recent article by Zeng et al. [17], artificial intelligence was used with whole-slide imaging of digital histological images of HCC to develop models that are able to predict the activation of six immune gene signatures. They achieved areas under the receiver operating characteristics curves in the validation dataset ranging from 0.81 to 0.92. However, this study focused on the prediction of tumours with upregulation of immune gene signatures with annotated cancer areas by a pathologist and gene signature status labels obtained via RNA sequencing. This study is in line with other studies using whole-slide imaging of available immunohistochemistry (IHC) stains or H&E in combination with state-of-the-art deep learning approaches, as these mostly investigate prognostic parameters with manual annotations of the cancer areas by pathologists [18,19,20].

The potential of multiplex images was also recently recognised in a study that used a multi-stain deep learning model (MSDLM) to predict prognoses and therapy responses in colorectal cancer. Single IHC stains of different immune cells were combined in the MSDLM to determine the AImmunoscore. The model had high prognostic capabilities and outperformed clinical, molecular, and immune-cell-based parameters. The authors stated that multiplexed images could potentially further improve the classification accuracy in terms of the prognosis and response to neoadjuvant therapy [18]. In addition, a number of articles have now been published that highlight the importance of multiplex imaging for cancers [21,22,23].

Pathologist-annotated tissue sections were used to analyse T cells and PD-L1^+^ cells in HCC patients. While PD-L1^+^ cells were present to a similar extent in PT and NTL tissues, significantly fewer CD3^+^ T cells were observed in the PT than in NTL tissues (3.9% vs. 8.1%; Table 3), which is consistent with previous studies analysing the landscape of HCC [24,25]. Furthermore, the HCC tumours were found to cultivate a suppressive immune system, as demonstrated by the enrichment of FoxP3^+^ cells (5.2% vs. 3.1%) and depletion of cytotoxic CD8^+^ cells (0.9% vs. 3.2%), which is in line with other studies [26,27]. In contrast to single-cell RNA sequencing studies that performed comprehensive cluster analysis [24,25], the HCC samples examined in this study based on multiplex immunofluorescence showed no enrichment of FoxP3^+^CD4^+^ cells in the PT tissues. It should be noted, however, that not all clusters examined showed enrichment of FoxP3^+^CD4^+^ regulatory T cells in the study by Lu et al. [24]. Furthermore, the number of detected cells generally depends strongly on the analysis method, e.g., RNA expression is not equivalent to protein expression, the investigated region(s) of interest, and the threshold for “positive” cells, among other things.

Based on the immunofluorescence images acquired with the TissueFAXS PLUS system and the newly developed software tool HCCMiner, 68 individual cell features were extracted for each cell (Figure 4, Table S1). The cell features showed strong heterogeneity between patients and within tumours. Subsequently, these features were used to investigate four different models (ICF, MANOVA, MLP, and U-net) for identifying the cancer area. Depending on the model, either all or a selection of the most important cell features were used based on their correlation with the tumour masks manually assigned by the pathologists. In addition, two normalisation methods were applied to the features before they were passed to the ICF, MANOVA, and MLP models. Model performance, which is typically assessed via accuracy (the ratio of correct predictions divided by all predictions) [28], did not improve with the applied normalisations (Table 4). The z-score-based normalisation performed even worse than no normalisation. Both neural networks, the MLP, and the U-net performed better compared with the correlation-based ICF and MONVA models (Figure 6, Table 4), with the U-net having the highest average accuracy of 75%. Furthermore, spatial representations of the cancer score matched well with the ground truth annotations of the pathologists (Figure 5). The U-net is a convolutional neural network (CNN) architecture specifically designed for biomedical image segmentation, which also takes into account the spatial context [13]. CNN architectures such as U-nets have recently shown their value in many medical applications, e.g., in detection, classification, and segmentation tasks based on radiological images [28,29], as they are robust to variations in shape, orientation, and position. Even though the achieved accuracy of 75% of the U-net seems low at first glance, it must be taken into account that this result was achieved with a limited number of samples (*n* = 12), a specific marker panel was used, and the calculation of the accuracy is based on the classification of each individual cell. The small number of HCC samples was also the reason why no subsequent prognostic analysis was performed based on the cancer and non-cancer areas in combination with, for example, individual markers, marker combinations, or different cell type densities. It is quite possible that there are markers that are more suitable for identifying the HCC area, such as CD34, cytokeratin (CK)7, or CK18 [27], and that have a high prognostic value in combination with other markers. Future work needs to identify these markers as well as the most suitable features while further optimising the neural network architecture, using a larger number of samples to reflect the heterogeneity of HCC observed in clinical practice. Predictions for the cancer area were not satisfactory for some samples. One reason for this could be that the ground truth annotations provided by the pathologists are relatively coarse (Figure 1). Therefore, refined ground truths in addition to larger sample sizes would potentially further improve the performance of the models. Another reason for the rather poor performance of the ICF and MANOVA approaches may be the partly contradictory marker expressions between some slides, which could potentially lead to the selection of suboptimal features. Both the ICF and the MANOVA methods select features that are highly correlated with the pathologists’ annotations, i.e., if multiple contradictory expression patterns are present in some slides, this could, in turn, decrease the correlation of the affected features even though they might perform well in some slides. The MLP and CNN approaches are more flexible in this regard and can theoretically learn multiple complex rules and feature combinations for improved performance. A consistency grouping of the data and training a set of dedicated models per group could potentially further improve the predictive capabilities. Moreover, a future avenue of research could be neural network architectures for point cloud processing such as PointNet [30] and its successors to directly perform predictions at the single-cell level as with the MLP approach while still having the ability to incorporate the spatial context of each cell as with the U-net. Finally, increasing the overall sample size of the analysed tissue sections can further improve the accuracy and generalisation capabilities of the learning-based approaches.

In conclusion, the results of this study indicate that convolutional neural networks such as the U-net are capable of learning to identify cancer areas based on multiplex immunofluorescence data. The spatial representations obtained with the computed cancer score delineate HCC well from non-cancer liver tissues. Future analyses with larger sample sizes will help to improve the model and allow us to calculate prognostic parameters based on the identified cancer areas in combination with cell features. The calculated prognostic parameters have great potential to facilitate the identification of low-risk patients and to support experts in decision making, e.g., on whether adjuvant therapy is necessary or which therapy should be selected.

## Figures and Tables

**Table 1:** Clinicopathological characteristics of the included HCC patients (*n* = 12).

Variable	Category	
Age at surgery (years)		Median: 69.0 (range 53.5–82.6)
Gender	Male	8 (66.7%)
	Female	4 (33.3%)
Tumour status (UICC7) *	pT1	3 (25.0%)
	pT2	4 (33.3%)
	pT3a	3 (25.0%)
	pT3b	1 (8.3%)
	pT4	0
	Not classified	1 (8.3%)
Regional lymph nodes (UICC7) *	pNX	12 (100.0)
Distant metastasis (UICC7) *	pM1	1 (8.3%)
Recurrence	No	8 (66.7%)
	Yes	4 (33.3%)
Vital status	Dead	9 (75.0%)
	Alive	3 (25.0%)

* According to the 7th edition of the International Union Against Cancer (UICC) staging system.

**Table 2:** List of monoclonal antibodies.

Antibody	Clone	Dilution	Incubation Time	Manufacturer
CD3	F7.2.38	1:1000	30 min	Dako
CD4	4B12	1:500	30 min	Dako
PD-L1	22C3	1:200	30 min	Dako
CD8	CD8/144B	1:500	30 min	Dako
FoxP3	PCH101	1:250	30 min	eBioscience

**Table 3:** Comparison of cells located in the hepatocellular carcinoma primary tumour (PT) tissues and in the adjacent/surrounding non-tumour liver (NTL) tissues. For this analysis, 10 randomly selected regions of interest, i.e., virtual biopsies, each with a size of 16 fields of view from both PT and NTL per sample (*n* = 12) were used to determine the mean percentage of “positive” cells for each marker and marker combination. Comparison with the Mann–Whitney U test between 978,069 cells located in PT and 765,976 cells in NTL. Statistically significant differences are marked with asterisks.

Lymphocyte Panel					PT	NTL	*p*-Value
CD3	CD4	CD8	FoxP3	PD-L1	Mean (SD) (%)	Mean (SD) (%)	
All “positive” cells for a given marker irrespective of the other markers (n. d. = not defined; pos. = positive).							
pos.	n. d.	n. d.	n. d.	n. d.	3.85 (4.23)	8.08 (6.48)	<0.001 *
n. d.	pos.	n. d.	n. d.	n. d.	2.83 (3.77)	6.14 (7.57)	0.023 *
n. d.	n. d.	pos.	n. d.	n. d.	0.90 (1.31)	3.21 (3.01)	<0.001 *
n. d.	n. d.	n. d.	pos.	n. d.	5.23 (9.05)	3.05 (5.22)	0.006 *
n. d.	n. d.	n. d.	n. d.	pos.	4.00 (6.89)	3.81 (6.30)	0.693
All possible marker combinations (pos. = positive; neg. = negative)							
neg.	neg.	neg.	neg.	neg.	86.26 (14.16)	82.19 (15.00)	0.022 *
pos.	neg.	neg.	neg.	neg.	2.46 (3.70)	4.30 (3.63)	<0.001 *
neg.	pos.	neg.	neg.	neg.	1.41 (1.98)	2.83 (3.58)	0.026 *
neg.	neg.	pos.	neg.	neg.	0.31 (0.44)	1.54 (1.58)	<0.001 *
neg.	neg.	neg.	pos.	neg.	4.14 (8.37)	1.81 (3.28)	0.002 *
neg.	neg.	neg.	neg.	pos.	2.78 (5.36)	2.07 (3.54)	0.598
pos.	pos.	neg.	neg.	neg.	0.61 (0.99)	1.66 (2.24)	0.006 *
pos.	neg.	pos.	neg.	neg.	0.22 (0.30)	0.82 (0.99)	<0.001 *
pos.	neg.	neg.	pos.	neg.	0.14 (0.19)	0.15 (0.22)	0.880
pos.	neg.	neg.	neg.	pos.	0.13 (0.33)	0.25 (0.44)	0.174
neg.	pos.	pos.	neg.	neg.	0.15 (0.42)	0.20 (0.30)	0.077
neg.	pos.	neg.	pos.	neg.	0.12 (0.31)	0.20 (0.38)	0.454
neg.	pos.	neg.	neg.	pos.	0.20 (0.56)	0.37 (0.77)	0.448
neg.	neg.	pos.	pos.	neg.	0.01 (0.05)	0.04 (0.08)	0.018 *
neg.	neg.	pos.	neg.	pos.	0.04 (0.10)	0.08 (0.17)	0.245
neg.	neg.	neg.	pos.	pos.	0.62 (1.13)	0.45 (1.16)	0.026 *
pos.	pos.	pos.	neg.	neg.	0.06 (0.12)	0.28 (0.44)	0.012 *
pos.	pos.	neg.	pos.	neg.	0.07 (0.13)	0.12 (0.21)	0.071
pos.	pos.	neg.	neg.	pos.	0.07 (0.17)	0.22 (0.44)	0.271
pos.	neg.	pos.	pos.	neg.	0.02 (0.10)	0.02 (0.05)	0.006 *
pos.	neg.	pos.	neg.	pos.	0.02 (0.06)	0.06 (0.11)	0.013 *
pos.	neg.	neg.	pos.	pos.	0.02 (0.08)	0.06 (0.21)	0.109
neg.	pos.	pos.	pos.	neg.	0.01 (0.08)	0.02 (0.04)	0.369
neg.	pos.	pos.	neg.	pos.	0.03 (0.12)	0.04 (0.10)	0.408
neg.	pos.	neg.	pos.	pos.	0.04 (0.10)	0.07 (0.19)	0.515
neg.	neg.	pos.	pos.	pos.	0.00 (0.01)	0.02 (0.06)	0.250
pos.	pos.	pos.	pos.	neg.	0.00 (0.01)	0.02 (0.05)	<0.001 *
pos.	pos.	pos.	neg.	pos.	0.01 (0.04)	0.05 (0.12)	0.011 *
pos.	pos.	neg.	pos.	pos.	0.03 (0.10)	0.05 (0.14)	0.032 *
pos.	neg.	pos.	pos.	pos.	0.00 (0.00)	0.01 (0.03)	0.017 *
neg.	pos.	pos.	pos.	pos.	0.00 (0.00)	0.01 (0.03)	0.030 *
pos.	pos.	pos.	pos.	pos.	0.00 (0.00)	0.01 (0.03)	0.016 *

**Table 4:** Comparison of the performance of the individual cell features (ICFs), the multivariate analysis of variance (MANOVA), and the multilayer perceptron (MLP) models with different normalisation methods and the U-net in terms of agreement with the pathologists’ assignments.

Method	Normalisation	Accuracy	Sensitivity	Specificity	Precision
ICF	None	0.65 (0.16)	0.82 (0.10)	0.49 (0.17)	0.65 (0.23)
	Feature-specific	0.65 (0.16)	0.78 (0.17)	0.51 (0.16)	0.64 (0.24)
	Z-score	0.63 (0.18)	0.90 (0.10)	0.27 (0.16)	0.61 (0.22)
MANOVA	None	0.65 (0.15)	0.81 (0.07)	0.52 (0.19)	0.65 (0.24)
	Feature-specific	0.63 (0.16)	0.75 (0.15)	0.51 (0.18)	0.63 (0.25)
	Z-score	0.64 (0.17)	0.91 (0.07)	0.32 (0.14)	0.61 (0.22)
MLP	None	0.69 (0.14)	0.66 (0.24)	0.67 (0.12)	0.66 (0.24)
	Feature-specific	0.70 (0.14)	0.66 (0.25)	0.67 (0.11)	0.66 (0.24)
	Z-score	0.67 (0.12)	0.77 (0.12)	0.60 (0.15)	0.69 (0.23)
U-net	None	0.75 (0.19)	0.62 (0.31)	0.87 (0.14)	0.75 (0.23)

Note: Accuracy computes how close a given set of results, in this case, the classification into either cancer or non-cancer tissue, are to their true values, i.e., the independent pathologists’ assignments. Sensitivity is the number of true positive results divided by the number of all results that should have been identified as positive. Sensitivity is the number of true negative results divided by the number of all results that should have been identified as negative. Precision is the number of true positive results divided by the number of all positive results, including those not identified correctly.

## Data Availability

The data presented in this study are available on request from the corresponding author.

## Supplementary Materials

The following supporting information can be downloaded at https://www.mdpi.com/article/10.3390/cells12071074/s1: Figure S1: Influence of individual cell features on the accuracy of the U-net. The plot shows the accuracies of the U-net, with one of the features omitted in each case. The model was trained and validated using eleven samples. The previously determined sample with the highest accuracy in identifying the cancer area was selected for testing. “without_area” means that the model was trained without the nucleus area feature, which has the strongest effect on the accuracy, as shown by the largest decrease in accuracy; Figure S2: Box plot showing the accuracy of the U-net for different region of interest (ROI) sizes. For each ROI size, the accuracy was determined by randomly selecting ten regions of interest (ROIs) each from the primary tumour and the non-tumour liver tissues. ClusterSize indicates the size of the randomly selected ROIs. For example, ClusterSize5 means that ROIs with a size of five fields of view have been selected. Full (Refined) means that all fields of view within a refined selection of the entire tissue section (exclusion of artefacts) have been analysed, and Full (Pathologist) that the whole slide has been analysed. Red lines indicate the medians, boxes the interquartile ranges (IQRs), and whiskers mark the respective minimum and maximum accuracy values for the ROIs; Figure S3: Influence of a cancer score threshold on the accuracy of the U-net. Plots show the accuracy (A), sensitivity (B), and specificity (C) of the U-net for different applied cancer score thresholds from 0 to 1, step size 0.05. Error bars mark the respective standard deviation; Table S1: The extracted features are summarized in the following table. In addition to the morphological (shape-related) features based on the detection and segmentation of the nucleus with DAPI, we also extracted intensity-based features for each of the six channels. These features comprise the mean intensity, the mean of the top 25% of the intensities, the maximum intensity, and the intensity standard deviation for each marker. In total, each cell is thus characterised by 37 basic features (in italics) and a user-defined selection of inferred features such as the number of active neighbours of each cell, the cell density, and the standard deviation (SD) of features in the neighbourhood of a cell; Table S2: Investigation of the influence of the size of the regions of interest (ROIs) on the accuracy, sensitivity, specificity, and precision. Primary tumour = PT, non-tumour liver tissue = NTL, and field of view = FOV.
