# Supplementary material for: A Deep-Learning-Computed Cancer Score for the Identification of Human Hepatocellular Carcinoma Area Based on a Six-Colour Multiplex Immunofluorescence Panel
Source: Cells. 2023 Apr 2;12(7):1074. doi: 10.3390/cells12071074 (PMC10093209; doi:10.3390/cells12071074)
Supplement: Supplementary file 1 [file cells-12-01074-s001.zip › cells-2272564-supplementary.pdf]

Supplementary Information

Table S1. The extracted features are summarized in the following table. In addition to the morphology (shape-related) features based on the detection and segmentation of the nucleus with DAPI, we also extracted intensity-based features for each of the six channels. These features comprise the mean intensity, mean of the top 25% of the intensities, the maximum intensity, and the intensity standard deviation for each marker. In total, each cell is thus characterised by 37 basic features (in italics) and a user-defined selection of inferred features such as the number of active neighbours of each cell, the cell density, and the standard deviation (SD) of features in the neighbourhood of a cell.

Parameter	Description
ID	*Unique integer identifier for each cell*
Area	*The number of pixels of the DAPI-based segmentation mask of each cell*
Centroid	*Centre of mass of the region (i.e., x, y coordinates)*
Circularity	*Value in range [0, 1] with 1 being a perfect circle*
Eccentricity	*Eccentricity of an ellipse with the same second-moments as the region. Feature values between 0 for circles, 1 for lines*
Equivalent Diameter	*Diameter of a circle with the same area as the nucleus region*
Extent	*Ratio of the area vs. the area of the bounding box*
Minor Axis Length	*Length of the minor axis*
Major Axis Length	*Length of the major axis*
Perimeter	*Perimeter of the region measured in pixels*
Solidity	*Ratio of the area of the region vs. the convex area*
Class Label	*Class labels "Tumour" vs. "Non-tumour" obtained from manual annotations by pathologists*
Mean Intensity	*Mean marker intensity averaged over all pixels of the DAPI-based segmentation mask*
Mean of the Top 25% of the Intensities	*Mean marker intensity of the brightest 25% pixels of the DAPI-based segmentation mask*
Maximum Intensity	*Maximum marker intensity of all pixels of the DAPI-based segmentation mask*
SD Intensity	*Standard deviation of the marker intensity of all pixels contained in the DAPI-based segmentation mask*
**Density**	Density of each nucleus is estimated as the number of neighbours in a circle with a user-defined radius. In all experiments we used r = 300 pixels
**Autofluorescence-corrected Marker Expression**	All intensity-based features can be postprocessed via an auto-fluorescence suppression and their expression can be classified. Assuming a Gaussian distribution of the intensity values, we compute the mean and SD values for each marker and use µ+2*sigma as the threshold value. All values exceeding this threshold are considered as "positive" cells and the remaining cells as "negative", i.e., not expressing the marker
**Number of Active Neighbours**	Counts the number of neighbouring cells within a user-defined radius that are categorised as active cells. In all experiments we used r=300 pixels
**Standard Deviation Features (SDF)**	For all features, the standard deviation (SD) can be computed. As for the density feature, we used a radius of 300 pixels to estimate the SD in the neighbourhood of each cell

Table S2. Investigation of the influence of the size of the regions of interest (ROIs) on the accuracy, sensitivity, specificity, and precision. Primary tumour = PT, non-tumour liver tissue = NTL, field of view = FOV.

Method	ROI size	Accuracy	Sensitivity	Specificity	Precision
Random selection of 10 ROIs in PT and NTL	1 FOV	0.69 (0.16)	0.58 (0.25)	0.82 (0.18)	**0.79 (0.18)**
	5 FOVs	0.70 (0.18)	0.61 (0.29)	0.76 (0.26)	0.75 (0.22)
	9 FOVs	0.66 (0.19)	0.59 (0.30)	0.72 (0.27)	0.70 (0.24)
	25 FOVs	0.69 (0.19)	**0.65 (0.32)**	0.71 (0.32)	0.75 (0.22)
Entire tissue section/whole slide		**0.75 (0.19)**	0.62 (0.31)	**0.87 (0.14)**	0.75 (0.23)

Note: *Accuracy* computes how close a given set of results, in this case the classification into either cancer or non-cancer tissue, are to their true value, i.e., the independent pathologists' assignment. *Sensitivity* is the number of true positive results divided by the number of all results that should have been identified as positive. *Sensitivity* is the number of true negative results divided by the number of all results that should have been identified as negative. *Precision* is the number of true positive results divided by the number of all positive results, including those not identified correctly.

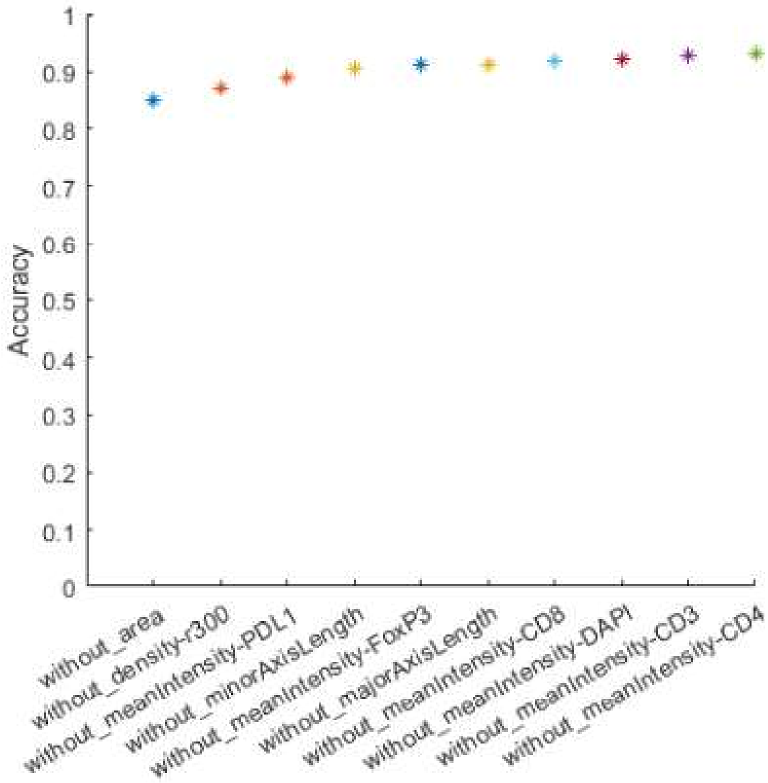

Figure S1. Influence of individual cell features on the accuracy of the U-net. The plot shows the accuracies of the U-net, with one of the features omitted in each case. The model was trained and validated using eleven samples. The previously determined sample with the highest accuracy in identifying the cancer area was selected for testing. "without_area" means that the model was trained without the nucleus area feature, which has the strongest effect on the accuracy, as shown by the largest decrease in accuracy.

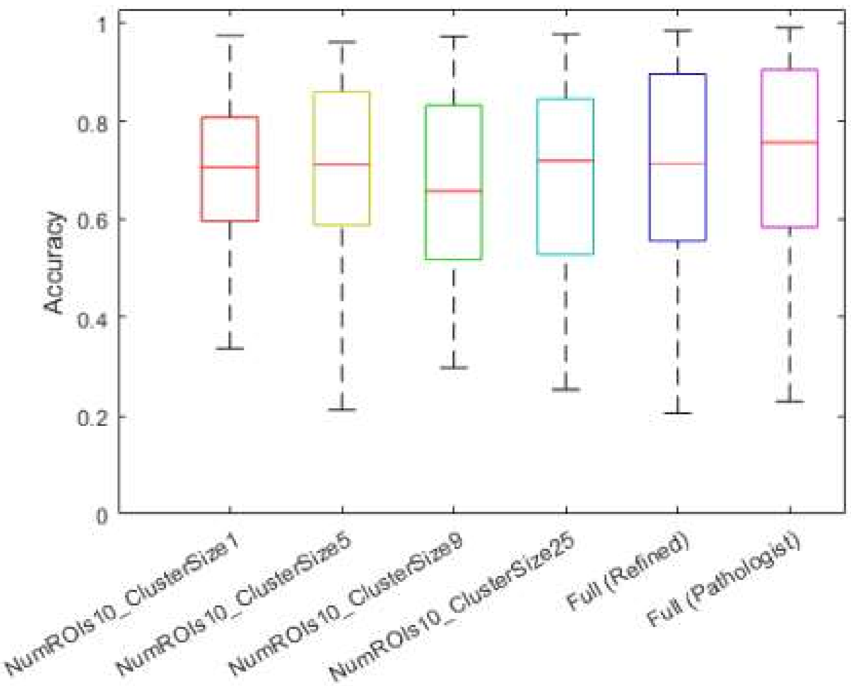

Figure S2. Box plot showing the accuracy of the U-net for different region of interest (ROI) sizes. For each ROI size, the accuracy was determined by randomly selecting ten regions of interest (ROIs) each from the primary tumour and the non-tumour liver tissues. ClusterSize indicates the size of the randomly selected ROIs. For example, ClusterSize5 means that ROIs with a size of five fields of view have been selected. Full (Refined) means that all fields of view within a refined selection of the entire tissue section (exclusion of artefacts) have been analysed, and Full (Pathologist) that the whole slide has been analysed. Red lines indicate the medians, boxes the interquartile ranges (IQRs), and whiskers mark the respective minimum and maximum accuracy values for the ROIs.

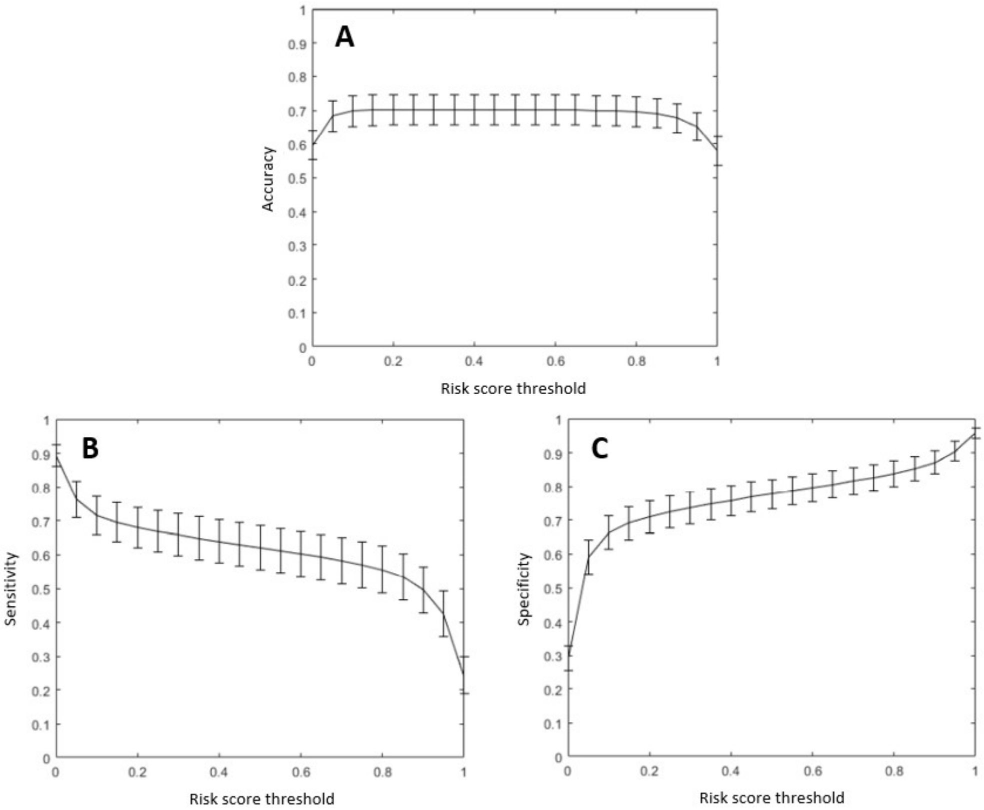

Figure S3. Influence of a cancer score threshold on the accuracy of the U-net. Plots show the accuracy (A), sensitivity (B), and specificity (C) of the U-net for different applied cancer score thresholds from 0 to 1, step size 0.05. Error bars mark the respective standard deviation.
